# Periodontal health status and lung function in two Norwegian cohorts

**DOI:** 10.1371/journal.pone.0191410

**Published:** 2018-01-19

**Authors:** Antonio Manuel Pérez Barrionuevo, Francisco Gómez Real, Jannicke Igland, Ane Johannessen, Ernst Omenaas, Karl A. Franklin, Laura Pérez Barrionuevo, Anne Nordrehaug Åstrøm, Cecilie Svanes, Randi Jacobsen Bertelsen

**Affiliations:** 1 Centre for International Health, Department of Global Public Health and Primary Care, University of Bergen, Bergen, Norway; 2 Department of Clinical Science, University of Bergen, Bergen, Norway; 3 Department of Global Health and Community Medicine, University of Bergen, Bergen, Norway; 4 Centre for Clinical Research, Haukeland University Hospital, Bergen, Norway; 5 Department of Surgical and Perioperative Sciences, Umeå University, Umeå, Sweden; 6 Mydentist, National Health Service, Bristol, United Kingdom; 7 Department of Clinical Dentistry, University of Bergen, Bergen, Norway; 8 Department of Occupational Medicine, Haukeland University Hospital, Bergen, Norway; University of Birmingham, UNITED KINGDOM

## Abstract

**Rationale and objectives:**

The oral cavity is united with the airways, and thus poor oral health may affect respiratory health. However, data on the interaction of periodontal and respiratory health is limited. We aimed to evaluate whether periodontal health status, assessed by the Community Periodontal Index (CPI), was related to lung function among young and middle-aged adults in two Norwegian cohorts.

**Methods:**

Periodontal health status and lung function were measured among 656 participants in the Norwegian part of the European Community Respiratory Health Survey (ECHRS III) and the RHINESSA offspring study. Each participant was given a CPI-index from 0 to 4 where higher values reflect poorer periodontal status. The association between CPI and lung function was estimated with linear regression adjusting for age, gender, smoking, body mass index, exercise, education, use of antibiotics, inhaled medication and corrected for clustering within families.

**Main results:**

Participants with CPI 3–4 had significantly lower FEV_1_/FVC ratio compared to participants with CPI 0, b (95% CI) = -0.032 (-0.055, -0.009). Poorer periodontal health was associated with a significant decrease in the FEV_1_/FVC ratio with an adjusted regression coefficient for linear trend b (95% CI) = -0.009 (-0.015, -0.004) per unit increase in CPI. This negative association remained when excluding asthmatics and smokers (-0.014 (-0.022, -0,006)).

**Conclusions:**

Poorer periodontal health was associated with increasing airways obstruction in a relatively young, healthy population. The oral cavity is united with the airways and our findings indicate an opportunity to influence respiratory health by improving oral health.

## Introduction

Poor periodontal health has been linked to many systemic conditions, including diabetes, cardiovascular and pulmonary diseases [1–3]. The relationship between periodontal and respiratory health is not well understood, but we know that the lungs are inhabited by bacteria that originate from the oral cavity [4]. The bacteria enters the lungs by microaspiration [4] or by systemic bacteria dissemination [5]. Furthermore, inflammatory mediators may be transferred in the blood from inflamed gingiva epithelium to other organs, such as the lungs [5]. Thus, there is a biological plaucible explanation for a link between periodontal health and lung health.

We have previously reported an association between self-reported gum bleeding and respiratory symptoms in a general population sample [6]. However, the majorities of the studies on the association between periodontal health and lung health have focused on elderly people and on chronic obstructive pulmonary disease (COPD) patients [7–10]. Chronic obstructive pulmonary disease has been associated with clinical markers of periodontitis [7, 9, 10] and tooth loss [11, 12]. Moreover, markers of periodontal disease are associated with reduced lung volumes and airflow limitation [8, 13]. Results from one intervention study and one randomized controlled trial, both on COPD patients with chronic periodontitis, found that periodontal therapy decreased exacerbation frequency [14, 15] and improved lung function [15]. However, these patients may suffer from periodontitis caused or aggravated by their COPD condition [16], and findings based on studies on patients with severe periodontitis are difficult to apply to the general population. The association of periodontal health with lung function in the general population is not well known.

A systematic review concluded that the incidence of severe periodontitis increased gradually from 30 to 80 years, but with a peak at 38 years of age [17], with considerable variance between regions and countries. Also younger age-groups suffer from periodontal problems in terms of poor oral hygiene, gingivitis and mild periodontitis [18]. In a WHO report based on 71% of the member states, it was found that dental caries and periodontal disease comprised a considerable public health problem in the majority of the countries [19], and the 2010 Global Burden of Disease Study estimated that severe periodontitis is the 6^th^ most prevalent disease worldwide, with an overall age-standardized global prevalence of 11.2% affecting around 743 million people [20].

We aimed to evaluate whether periodontal health status, assessed with the Community Periodontal Index (CPI), was related to lung function among relatively young Norwegian adults from a general population sample. By gaining more knowledge on the associations between periodontal health and lung health in young and middle-aged adults, this may ultimately provide a rational for oral intervention programs to prevent development and progression of chronic respiratory diseases. The Community Periodontal Index is the reference method used for the WHO Global Oral Data Bank [21]; it is a standardized and validated clinical tool used to evaluate periodontal status [22].

## Material and methods

### Study design and participants

The present study included Norwegian participants in the RHINESSA generation study (Respiratory Health in Northern Europe, Spain and Australia; www.RHINESSA.net) and the third wave of the ECRHS study (European Community Respiratory Health Survey; www.ecrhs.org). ECRHS [23] is an international survey initiated in 1992–1994 which recruited randomly selected individuals aged 20–44 years from general populations in more than 36 centres in 16 countries. The participants were invited to follow-up studies, ECRHS II and III, which took place in 1998–2002 and 2010–13. The RHINESSA study investigates the offspring of ECRHS participants in ten centres in seven countries. At the ECRHS and the RHINESSA surveys the examinations included an interviewer-led questionnaire, lung function measurements, and blood samples for measurement of serum specific IgEs.

The present analysis includes data from the Norwegian study centre in Bergen, where an additional investigation of oral health was added to the main protocol for both parents and offspring. The study population consists of 247 ECRHS III participants in 2012–13 and 409 RHINESSA participants in 2014, who had measurements of both lung function and CPI.

Ethical approval was obtained from the Regional Committee for Medical and Health Research Ethics in Western Norway (approval numbers #2010/759 and #2012/1077). All participants provided informed written consent prior to study participation.

### Lung function

Lung function was measured using a standard spirometric method [24]. The maximum forced expiratory volume in one second (FEV_1_) and maximum forced vital capacity (FVC) of up to five technically acceptable manoeuvres were selected, even if they did not come from the same manoeuvre, and the FEV_1_/FVC ratio was calculated from these. Height and weight and CPI were measured by the field worker before measurement of lung function. In addition, we calculated percent predicted values for FEV_1_ and FVC based on the method and reference values reported in Johannessen *et al* [25].

### Community Periodontal Index (CPI)

According to WHO guidelines, CPI is used to screen/assess the periodontal status [26]. CPI was measured using a standardized protocol to investigate: gum bleeding, calculus and periodontal pockets. The following ten teeth: Upper right; 17 and 16; upper anterior: 11; upper left: 26 and 27; lower right: 47 and 46; lower anterior: 31; and lower left: 36 and 37 were examined and each tooth was given its highest CPI score ranging from 0 to 4.

0: healthy periodontal status
1: bleeding on probing
2: calculus detected during probing
3: shallow periodontal pockets (4–5 millimeters)
4: deep periodontal pockets (≥6 millimeters)

To record the CPI the probe was led around the sulcus of the buccal side of the index teeth in each sextant. For each individual, the CPI score was defined as the maximum score obtained across all 10 index teeth [26]. The CPI measures were performed without saliva control with the patient lying down on an examination bed as we did not have access to a dental chair. The CPI measures were performed by two study nurses trained and calibrated by a dentist. The protocol was based on the WHO recommendation and verified by a dentist.

### Covariates

Data on sociodemographic factors, lifestyle and general health were obtained from interviews. Body mass index (BMI) was calculated from measured weight and height. Gastro-esophageal reflux disease (GERD) was defined as having heartburn or belching 3–5 nights/week or more. Nasal congestion was defined as having a blocked nose >12 weeks during the last 12 months. Participants were defined as atopic if they had specific IgE ≥0.35 kU/L for at least one of the allergens: cat, timothy or house dust mite (HDM) [27]. Atopy information was unavailable for 106 participants. Other variables were defined according to questions described S1 Appendix and provided at www.ecrhs.org and www.rhinessa.net.

### Statistical analyses

Descriptive statistics for the study population was reported as mean and standard deviation for continuous variables and numbers and percentages for categorical variables. Linear trend in characteristics across the ordinal CPI categories were tested using linear regression, logistic regression and multinomial logistic regression with CPI included as a continuous variable from score 0 to 4. The lung function measures, FVC, FEV_1_ and FEV_1_/FVC, were modelled as continuous dependent variables in linear regression models. CPI was included both as a categorical variable with score 0 as the reference category and as a continuous variable to test for a linear trend. In regression models using CPI as a categorical variable with three categories; score 3 and 4 were collapsed into one group because of very few individuals with score 4. Results from linear regression were reported as unstandardized regression coefficients, b, with 95% confidence intervals and p-value for linear trend across categories of CPI.

Three different levels of adjustment are presented. In Model 1 we adjusted for age and sex. Models with FVC and FEV_1_ as the dependent variables also included height, interaction between height and sex and between age and sex. Model 2 included all variables in Model 1 plus additional adjustment for smoking. Model 3 included all variables in Model 2 plus additional adjustments for BMI, exercise and education. All models were tested for interaction between CPI and smoking and between CPI and gender, but significant interactions were not found. In all regression models standard errors were corrected for clustering within families (parent-offspring and siblings) by applying a clustered sandwich estimator (accounting for the fact that family members are not independent from each other).

Various sensitivity analyses of the association between FEV_1_/FVC ratio and CPI adjusted as in Model 3 were performed and included models stratified by gender and by cohort (parents, offspring). One model excluded all persons with doctor-diagnosed asthma (ever) and all persons who had been on asthma medication or antibiotics during the last 12 months to help breathing, in addition to one model excluding smokers and former smokers. We also constructed a model for the total study population with additional adjustment for frequency of tooth brushing in addition to the variables specified in model 3 and models stratified by atopic status. Stratification by atopy was included to account for potential confounding by rhinitis and blocked nose which has been associated with poor oral health. Finally, we constructed models with CPI, calculated as the mean of the score, for the ten index teeth for each individual, rather than using the maximum, in order to see whether use of the maximum caused any loss of important variation.

STATA (StataCorp, College Station, TX, USA), version IC 14.0 was used in all analyses.

## Results

### Population characteristics according to CPI

Of the 656 participants, 483 (76.6%) had a CPI score of 0, indicating healthy periodontium, and 56 (8.6%) had score 3 or 4 indicating mild to moderate and severe periodontal disease (Table 1). The mean age of the participants was 37.5 years and age increased significantly with increasing CPI score (p<0.001). A higher percentage of men than women had CPI score ≥1. Smoking and low education was also significantly associated with higher CPI scores (Table 1). There was no significant trend across categories of CPI for frequency of tooth brushing, BMI, exercise, GERD, asthma diagnosis, problems with blocked nose, atopy or unspecific course of antibiotics in the last 12 months, while use of inhaled medication as well as antibiotics to help breathing were positively associated with increasing CPI scores (p<0.001). There was no trend in mean FVC-levels across the categories of CPI, but a significant trend in mean FEV_1_ and mean for the ratio of FVC to FEV_1_ was observed in both men and women (Table 1). No significant trend was observed for FEV_1_ and FVC when reported as percent predicted (Table 1).

**Table 1:** Descriptive analysis by CPI of study population.

Variables	Total	CPI					p-trend*
		Score 0	Score 1	Score 2	Score 3	Score 4	
**N** (%)	656	483 (76.6)	38 (5.8)	79 (12.0)	51 (7.8)	5 (0.8)	
**Age,** mean (sd)	37.5 (13.8)	35.5 (13.4)	41.8 (14.8)	41.3 (12.8)	45.9 (12.8)	52.9 (6.5)	<0.001
**Sex,** n (%)							
Men	342	223 (65.2)	26(7.6)	62 (18.1)	28 (8.2)	3 (0.9)	
Women	314	260 (82.8)	12 (3.8)	17 (5.4)	23 (7.3)	2 (0.6)	<0.001
**Education**							
Primary	29	14 (48.3)	3 (10.3)	5 (17.2)	5 (17.2)	2 (6.9)	
Secondary	277	204 (73.7)	13 (4.7)	41 (14.8)	17 (6.1)	2 (0.7)	
University/College	348	263 (75.7)	22 (6.3)	33 (9.5)	29 (8.3)	1 (0.3)	<0.001
**Smoking habit**							
Never smoker	391	311 (79.5)	18 (4.6)	39 (10.0)	23 (5.9)	0 (0)	
Former smoker	159	116 (73.0)	13 (8.2)	17 (10.7)	13 (8.2)	0 (0)	
Current smoker	101	53 (52.5)	7 (6.9)	23 (22.8)	14 (13.9)	4 (4.0)	<0.001
Packyears of smoking, mean (sd)**	3.3 (9.6)	2.1 (9.6)	4.2 (7.3)	6.5 (12.8)	7.7 (17.6)	19.5 (19.8)	0.001
Packyears (smokers and ex-smokers) mean (sd)	11.4 (15.1)	8.9 (12.9)	9.7 (12.9)	16.1 (16.0)	19.5 (23.9)	19.5 (19.8)	0.009
**Frequency of tooth brushing**							
< 2 times per day	136	101 (74.3)	5 (3.7)	18 (13.2)	11 (8.1)	1 (0.7)	
≥ 2 times per day	519	382 (73.6)	33 (6.4)	60 (11.6)	40 (7.7)	4 (0.77)	0.86
**BMI,** mean (sd)	25.8 (4.7)	25.6 (4.8)	26.7 (4.4)	26.9 (4.4)	25.4 (4.2)	26.8 (6.1)	0.32
**Exercise**							
Never	24	14 (58.3)	2 (8.3)	6 (25.0)	1 (4.2)	1 (4.2)	
< once per week	103	72 (69.9)	8 (7.8)	12 (11.7)	10 (9.7)	1 (1.0)	
once a week	123	88 (71.5)	10 (8.1)	10 (8.1)	14 (11.4)	1 (0.8)	
2–3 times per week	267	195 (73.0)	10 (3.8)	39 (14.6)	21 (7.9)	2 (0.8)	
Almost every day	135	113 (83.7)	7 (5.2)	10 (7.4)	5 (3.7)	0 (0)	0.003
**GERD**^¤^**, n** (%)							
No	609	451 (74.1)	36 (5.9)	73 (12.0)	46 (7.6)	3 (0.5)	
Yes	43	30 (69.8)	2 (4.7)	6 (14.0)	4 (9.3)	1 (2.3)	0.35
**Ever diagnosed with asthma by a doctor**						
No	524	395 (75.4)	27 (5.2)	59 (11.3)	39 (7.4)	4 (0.8)	
Yes	120	80 (66.7)	10 (8.3)	20 (16.7)	9 (7.5)	1 (0.8)	0.15
**Problems with blocked nose last 12 months**						
No	368	273 (74.2)	20 (5.4)	45 (12.2)	27 (7.3)	3 (0.8)	
Yes	278	205 (73.7)	17 (6.1)	34(12.2)	20 (7.2)	2 (0.7)	0.99
**Atopy-positive**							
No	350	266 (76.0)	19 (5.4)	37 (10.6)	26 (7.4)	2 (0.6)	
Yes	200	156 (78.0)	10 (5.0)	26 (13.0)	8 (4.0)	0 (0)	0.32
**Use of antibiotics last 12 months to help breathing**						
No	553	416 (75.2)	31 (5.6)	59 (10.7)	45 (8.1)	2 (0.4)	
Yes	46	25 (54.4)	4 (8.7)	10 (21.7)	5 (10.9)	2 (4.4)	0.002
**Used inhaled medicines to help breathing at any time in the last 12 months**						
No	553	421 (76.0)	29 (5.2)	61 (11.0)	40 (7.2)	2 (0.4)	
Yes	98	60 (61.2)	9 (9.2)	17 (17.4)	10 (10.2)	2 (2.0)	0.003
**FEV**_**1**_**, mean**(sd)							
Men	4.2 (0.8)	4.2(0.7)	4.1 (0.9)	4.1 (0.8)	3.8 (0.8)	3.4 (1.7)	0.007
Women	3.1 (0.6)	3.2 (0.6)	2.7 (0.8)	3.0 (0.4)	3.0 (0.7)	2.1 (0.2)	0.02
**FEV**_**1**_**%-predicted, mean (sd)**	95.4 (17.3)	95.8 (17.3)	93.4 (17.7)	95.6 (14.2)	93.4 (19.6)	87.3 (31.7)	0.30
**FVC, mean**(sd)							
Men	5.3 (0.8)	5.3 (0.8)	5.3 (1.1)	5.3 (0.8)	5.1 (0.9)	5.2 (1.0)	0.41
Women	3.9 (0.7)	3.9 (0.7)	3.4 (0.7)	4.0 (0.7)	4.0 (0.8)	2.7 (0.2)	0.60
**FVC %-predicted, mean (sd)**	98.2 (17.0)	97.7 (17.1)	96.6 (16.1)	100.5 (15.9)	100.7 (18.6)	99.8 (20.6)	0.12
**FEV**_**1**_**/FVC, mean**(sd)							
Men	0.79 (0.08)	0.80 (0.07)	0.79 (0.07)	0.77 (0.06)	0.74 (0.10)	0.64 (0.23)	<0.001
Women	0.80 (0.09)	0.81 (0.09)	0.78 (0.16)	0.76 (0.07)	0.75 (0.07)	0.77 (0.00)	<0.001

*Estimated using linear regression for continuous variables and logistic and multinomial logistic regression for categorical variables with the CPI-index included as a continuous variable in the model. Corrected for clustering within families by using the clustered sandwich estimator for estimation of standard errors in Stata.

**n = 108 (16.5%) with missing information for numbers of years with smoking.

^¤^GERD: Gastroesophageal reflux disease

### CPI and lung function

The age-and sex-adjusted linear regression model with FEV_1_ as the dependent variable and CPI as a categorical variable showed no significant differences between CPI 0 and the other categories (Table 2). The model with test for linear trend showed borderline significant trend over CPI categories (p = 0.07), with b (95% CI) = -0.046 (-0.101, 0.003) change in FEV_1_ per unit increase in CPI. After further adjustments for smoking, BMI, exercise and education the association weakened. A positive association was indicated for FVC with increasing CPI index, but this did not reach statistical significance (Table 2). Pack-years were strongly associated with CPI (Table 1), but due to the large number of participants with missing information (16%), we chose to include the 3-category smoking variable in our main analyses. However, in a sensitivity analyses with adjustment for pack-years as a continuous variable instead of the 3-category smoking-variable in the model with FEV_1_/FVC ratio as outcome, the regression coefficient was slightly reduced from -0.009 to -0.008, but the association remained significant (95% CI: -0,015, -0.001).

**Table 2:** Association between lung function and CPI by logistic regression (CPI in categories) and linear regression (CPI as continuous variable).

	Categorical CPI				Continuous CPI	
	Score 0	Score 1 b (95%CI)*	Score 2 b (95%CI)*	Score 3–4 b (95%CI)*	b(95% CI)**	p-trend
**FEV1**						
Model 1: Age, sex, height, height*sex, age*sex	ref	-0.086 (-0.299, 0.127)	-0.037 (-0.165, 0.092)	-0.173 (-0.364, 0.018)	-0.046 (-0.101, 0.003)	0.07
Model 2: Age, sex, height, height*sex, age*sex, smoking	ref	-0.073 (-0.288, 0.142)	-0.010 (0.141, 0.121)	-0.155 (-0.346, 0.036)	-0.038 (-0.088, 0.013)	0.14
Model 3: Age, sex, height, height*sex, age*sex, smoking, bmi, exercise, education	ref	-0.088 (-0.295, 0.118)	-0.018 (-0.150, 0.113)	-0.130 (-0.305, 0.045)	-0.033 (-0.080, 0.014)	0.17
**FVC**						
Model 1: Age, sex, height, height*sex, age*sex	ref	-0.075 (-0.330, 0.180)	0.053 (-0.103, 0.209)	-0.019 (-0.232, 0.194)	0.002 (-0.054, 0.058)	0.94
Model 2: Age, sex, height, height*sex, age*sex, smoking	ref	-0.063 (-0.321, 0.195)	0.074 (-0-084, 0.232)	-0.013 (-0.232, 0.205)	0.007 (-0.048, 0.066)	0.81
Model 3: Age, sex, height, height*sex, age*sex, smoking, bmi, exercise, education	ref	-0.071 (-0.318, 0.176)	0.062 (-0.094, 0.217)	0.014 (-0.194, 0.222)	0.012 (-0.044, 0.067)	0.68
**FEV1/FVC**						
Model 1: Age, sex	ref	-0.001 (-0.035, 0.033)	-0.015 (-0.031, 0.001)	-0.037 (-0.062, -0.012)	-0.010 (-0.017,- 0.004)	0.002
Model 2: Age, sex, smoking	ref	0.000 (-0.034, 0.034)	-0.013 (-0.029, 0.003)	-0.034 (-0.058, -0.010)	-0.009 (-0.003, -0.002)	0.004
Model 3: Age, sex, smoking, bmi, exercise, education	ref	-0.001 (-0.036, 0.033)	-0.013 (-0.029, 0.004)	-0.032 (-0.055, -0.009)	-0.009 (-0.015, -0.004)	0.004

*Regression coefficients with 95% confidence intervals

**Regression coefficients with 95% confidence intervals per unit change in CPI. Corrected for clustering within families by using the clustered sandwich estimator for estimation of standard errors in Stata

**Fig 1:**
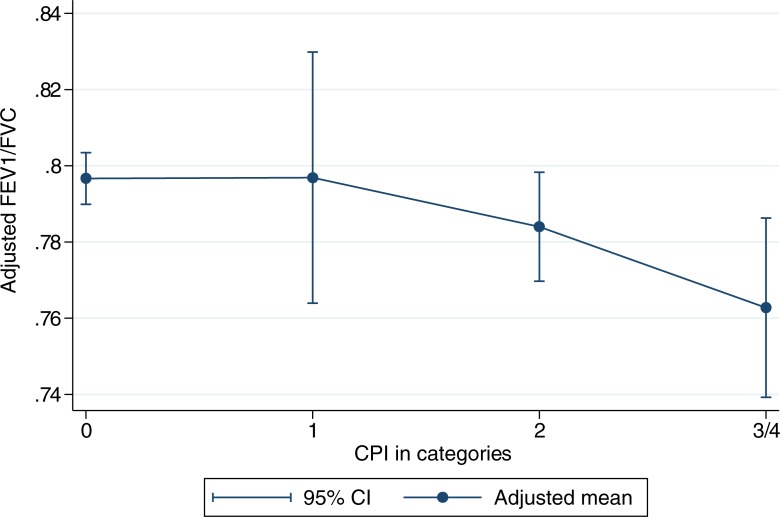
Adjusted FEV_1_/FVC ratio in categories of CPI with category 3 and 4 combined. Adjusted for age, sex and smoking.

Participants with CPI score 3 or 4 had significantly lower FEV_1_/FVC ratio compared to participants with score 0 (Table 2). In addition, there was a significant negative linear association between higher CPI index and lower FEV_1_/FVC ratio in all models, consistent with adjustments for a range of factors (Fig 1 and Table 2).

In the gender-stratified model for FEV_1_/FVC ratio (Model 4) we found a strong significant negative linear trend in men, and a weaker non-significant negative association in women, although there was no significant interaction between CPI and gender (Table 3).

**Table 3:** Sensitivity analyses for the association between FEV_1_/FVC ratio and CPI in different subgroups.

	Model specification	n	B(95% CI)	p-trend
Model 4	By gender			
	Men	338	-0.012 (-0.020, -0.003)	0.008
	Women	307	-0.005 (-0.013, 0.004)	0.31
Model 5	By cohort			
	Parent	238	-0.007 (-0.016, 0.001)	0.09
	Offspring	407	-0.010 (-0.018, -0.001)	0.023
Model 6	Persons without asthma or medication last 12 months to help breathing	478	-0.010 (-0.016, -0004)	0.001
Model 7	Never-smokers without asthma or medication last 12 months to help breathing	308	-0.014 (-0.022, -0.006)	0.001

Models adjusted for age, sex (models 5–7), smoking (models 4–6), BMI, exercise and education. Corrected for clustering within families by using the clustered sandwich estimator for estimation of standard errors in Stata

The model stratified by cohort did not show marked differences between parents and offspring (Model 5); the association appeared to be slightly stronger in the offspring cohort but there was no significant interaction between CPI and cohort (Table 3).

We restricted the model to never-smokers who had never been diagnosed with asthma and had not used antibiotics or inhaled medication to help breathing in the last 12 months, and the association between CPI and FEV_1_/FVC was even stronger with b (95% CI) = -0.014 (-0.022, -0.006) decrease in FEV_1_/FVC per unit increase in CPI (Model 7, Table 3). However, interaction-tests did not reveal any significant interaction with smoking or medication. Additional adjustment for frequency of tooth brushing did not change the association between CPI and FEV_1_/FVC; b (95% CI) = -0.009 (-0.015, -0.003).

Atopy was found in 200 (36%) of 550 participants who contributed with IgE measurement. Among the 350 non-atopic participants, there was a statistically significant negative association between increasing CPI and FEV_1_/FVC, with b (95% CI) = -0.008 (-0.018, -0.002) decrease in FEV_1_/FVC per unit increase in CPI after adjustment for age and sex. Among the 200 atopic subjects, the regression coefficient was the same as for the non-atopic subjects, but not significant b (95% CI) = -0.008 (-0.016, -0.001), which could be due to the smaller sample size.

Results from analyses with mean CPI rather than maximum CPI scores and FEV_1_/FVC ratio are shown in S1 Table and S1 Fig. The correlation between mean CPI and maximum CPI was strong (r = 0.75) and the association with FEV_1_/FVC was still present and strengthened after restriction to never-smokers without asthma or medication to help breathing the last 12 months, in line with the findings for maximum CPI.

## Discussion

In this population-based study we found that a commonly used measure of airways obstruction, the FEV_1_/FVC ratio, was associated with periodontal health status measured by CPI. The association of the FEV_1_/FVC ratio with CPI was consistent after adjustments for a range of potential confounding variables such as age, gender, educational level, smoking habits, and tooth brushing. Further, use of inhaled asthma medication, which could potentially influence periodontal health, did not explain the association; as seen by the equally strong association after excluding asthmatics and persons using asthma medication.

To the best of our knowledge, no previous study has assessed associations between periodontal status measured by CPI and lung function per se. One study from Korea measured CPI and used spirometry to define COPD status [28]. They found the more severe periodontal status (CPI 3 or 4) to be more common among male COPD patients. The Korean adults (>40 years of age) had poorer periodontal status than in our population (26% and 11% with CPI index score of 3 and 4, respectively, compared to 7.8% and 0.8% in the present study). In the present study we did not find any significant effect modification by gender. Their and our findings generally agree in supporting a link between poor periodontal health and respiratory health as reported in the literature. In a multi-center study report, which included the parent cohort in the current study, we have previously reported associations between self-reported gum bleeding and respiratory symptoms [6]. Katancik *et al*. reported an association of periodontal disease measured by gingival index and loss of attachment with airways obstruction measured by the FEV_1_/FVC ratio in a population of 70–79 year old participants [8]. In the NHANES study (with participants of similar age-range as in the present study), it was found that the FEV_1_/FVC ratio appeared to diminish with increasing periodontal attachment loss [29]. Although we used a different clinical marker for periodontal status (pocket depths instead of attachment loss), our study supports the findings of these two previous studies [8, 29] on associations between poor periodontal health and FEV_1_/FVC ratio. In a general adult population from Poland, periodontal disease was significantly associated with reduced lung volumes and airflow limitation [13]. Similar to our study, the associations were also seen after restricting the analysis to never-smokers. In the present study, a higher CPI score was more common for current smokers as compared to never-smokers, and also strongly associated with pack-years. It is well-known that smoking affects periodontal health [30] and evidence for the association between smoking and chronic periodontitis has been demonstrated in diverse populations [31]. One study found that the association between poor periodontal health and lower lung function was partly explained by the detrimental effects of smoking [32]. In a review paper by Garcia et al, concluding that epidemiological evidence indicates that poor periodontal health status is associated with increased risk of COPD, the authors also suggested that confounding by tobacco smoking may in part account for the association [33]. Although we assessed lung function and not COPD, we found that in the subgroup of never-smokers, the association between CPI scores and FEV_1_/FVC was still significant and even slightly stronger compared to the model with smokers included. Also in studies on COPD, associations between periodontal disease and COPD have been reported for non-smokers [8, 10].

In the present study, we collected information on frequency of tooth brushing, and in addition also information about frequency of use of fluoride tooth paste, mouth wash, tooth picks and dental floss. Frequency of use of dental floss, tooth picks and mouth wash did not differ by CPI score. The use of fluoride tooth paste correlated with the frequency of tooth brushing, as there are very few who do not use fluoride tooth paste [34]. The variability of tooth brushing frequency was low and not associated with the CPI score. After adjustment for tooth brushing with three or four frequency categories instead of only two (see S1 Appendix for information on categories), the regression coefficient for CPI remained unchanged.

Periodontal health appears to have improved among young adults in Norway. The prevalence of 35-year olds in Oslo having CPI score 4 declined from 21.8% in 1984 to 8.1% in 2003 [35]. The prevalence is lower (CPI score 4 = 0.8%) in our study, possibly indicating further improvement in periodontal health. But the different cohorts, geographical areas and study periods make it difficult to directly compare the groups. Our study participants may represent a healthy selection of the general population. It is interesting to note that despite the relatively healthy population sample, we still observe an association between increasing CPI score and reduced lung function. This is important in a public health perspective, as it might indicate that early oral intervention to inhibit progression towards severe periodontal disease might be beneficial for lung health.

The present study includes one population (ECRHS) recruited as a population-based sample, and their offspring which are participants in the RHINESSA study, initiated 20 years after the ECRHS was established. It is likely that clustering of disease in close family members may be explained by sharing of either environmental or genetic factors or both. Parents with poor periodontal health tend to have offspring with poor periodontal health [36]. With regard to lung function, little is known about heredity between parents and offspring. It has been suggested that non-specific bronchial hyper-reactivity (BHR) has an autosomal dominant pattern of inheritance [37] and that BHR, regardless of asthma status, increases the risk of offspring asthma [38, 39]. In the present study, the two cohorts have different age ranges, which makes it difficult to compare periodontal health status and spirometry measures between the two groups, as both measures will be age-dependent. However, in our analyses, the family structure is accounted for in the statistical analyses, with correction for clustering within families. Adjustment for clustering takes into account that individuals within each family is more similar and closely related (not statistically independent) than independent individuals. In addition, we observed approximately the same results in analyses done separately for the two generations. Thus, the association between periodontal status and lung function are unlikely to be exaggerated due to the familiar link.

Poor periodontal health is more frequently reported for patients with rhinitis [40] and it is hypothesized that this may be due to xerostomia occurring with blocked nose, potentially making a favourable environment for bacteria causing periodontitis. Some bacteria and micro-organisms are transferred from the oral cavity to the lungs, and thus pathogens inhabiting the oral cavity, for instance those associated with periodontal disease, may possibly impact respiratory health directly or indirectly through systemic inflammation, or drive common immunological components that affect epithelia in both periodontal and respiratory tissues [41]. In the present study, however, we did not see a link between blocked nose, symptoms of rhinitis or atopy and CPI score.

Asthma medication may have side effects on oral health [42], and it has been discussed whether the association between poor oral health and respiratory symptoms are due to reverse causation, with the lung disease or medication causing poor periodontal health [43]. This has not been properly addressed in previous studies on periodontal and respiratory disease. However, in the present study, as well as in our previous report on gum bleeding [6], medication did not explain the association of CPI and gum bleeding with airway disease and obstruction. Moreover, among never-smokers who had not taken any asthma or inhaled medications in the last 12 months, we found an even stronger negative association between CPI and the FEV_1_/FVC ratio.

In the analysis of lung function and CPI, we observed a consistent association of CPI with lower FEV_1_/FVC ratio, resulting from a (borderline significant) negative association with FEV_1_ and a slightly positive association between CPI and FVC. The absolute magnitude of the effects is relatively small. We suggest that the findings tell more about an underlying pathophysiological process, rather than being directly clinically relevant on a group level. The fact that the association was consistent and significant for airways obstruction–the FEV_1_/FVC ratio–rather than for FEV_1_, is of particular interest, as this might indicate an underlying biological mechanism that could involve an ongoing inflammatory process rather than a process related to growth and development. This gives some optimism with regard to potential for prevention–which certainly goes beyond the scope of the current analysis, and needs to be investigated in future studies.

### Strengths and limitations

The cross-sectional design of our study makes it difficult to determine a cause-effect relationship between periodontal health and lung function. Nevertheless, adjustment for a range of potential confounding variables and sub-group analyses did not attenuate the association between CPI and FEV_1_/FVC.

In this study we used CPI and measures of lung function which are both standardized and recommended methods for measuring periodontal status and respiratory health, respectively. CPI may however provide misleading periodontal status in a person with few representative teeth. The population in the present study was relatively young and thus we did not have the problem of missing index teeth. As with all existing periodontal disease measurement tools, the CPI index has advantages and limitations [44]. The CPI tool has been criticized for not taking clinical attachment loss into account, which might contribute to some underestimation of periodontal disease [44]. The CPI score has also been criticized for the difficulty of translating the index into treatment needs. However, the intention with the present study was not to document treatment needs, but rather to define periodontal health status with a tool that has been accepted and recommended and that enable comparison with other populations and studies using the same instrument. Moreover, we also considered the mean CPI of all the measured index teeth, but still found a statistical significant negative linear association between mean CPI and FEV_1_/FVC, similar to the results of the models applying the maximum CPI. In our study CPI measures were performed without use of dental chair and saliva control which would be ideal. However, given that all examinations were performed under equal conditions, this is unlikely to have impact on the associations described in this study.

The study population in the present study had a high level of education, with 53% reporting university or college degree. Presently, 33% of the Norwegians have university or college education [45]. It is well known that persons who volunteer to participate in health surveys are healthier and have higher education than the general population. In another paper from a large-population based Norwegian study with high educational level among the participants [46], the authors concluded that the over-representation of women with high education resulted in biased prevalence-estimates but little or no bias in exposure-outcome associations. Thus, overrepresentation of participants with high education in our study may have resulted in lower prevalence of periodontal disease and better lung function than the general population, but it is unlikely that the association between CPI and lung function is biased by this selection. Nevertheless, having adjusted for educational level in all our analyses and despite the healthy, relatively young and well-educated population included in the present study, this makes our findings even more important in a global public health perspective [47].

### Conclusion

Poor periodontal health is related to airways obstruction. The reported association between CPI and the FEV_1_/FVC ratio supports the concept that the continuum from the oral cavity and to the lungs is important, and that the oral cavity should be included in the “united airways” perspective. The potential to prevent or reduce future respiratory disease by improving oral hygiene may provide an important impact on public health.

## Supporting information

S1 Appendix (DOCX)

S1 Fig Graph box of mean CPI per person over categories of maximum CPI per person. (EPS)

S1 File Data file with variables. (XLSX)

S1 Table Association between FV1/FVC-ratio and mean CPI, linear regression. (DOCX)
