# Supplementary material for: Periodontal health status and lung function in two Norwegian cohorts
Source: PLoS One. 2018 Jan 19;13(1):e0191410. doi: 10.1371/journal.pone.0191410 (PMC5774767; doi:10.1371/journal.pone.0191410)
Supplement: S1 Appendix — (DOCX) [file pone.0191410.s001.docx]

**S1 Appendix for “Poor periodontal health and airways obstruction in two Norwegian cohorts”**

**Material and methods**

**Covariates**

Data on sociodemographic factors, lifestyle and general health were obtained from a mixture of self-administered questionnaires and clinical interviews done the same day as the measurement of lung function. Smoking history was assessed by the questions *“Have you ever smoked for as long as a year?”* and *“Do you now smoke, as of one month ago?”*; defining never smokers, current smokers and ex-smokers. Asthma was defined according to the question *“Have you ever been diagnosed with asthma by a doctor? “*. Use of asthma medication and antibiotics were assessed by the questions *“Have you used inhaled medicines to help breathing at any time in the last 12 months?”* and *“Have you had a course of antibiotics in the last 12 months to help your breathing?”* Frequency of tooth brushing was assessed by the question “*How often do you usually brush your teeth*?” The answer categories for tooth brushing for the ECRHS study were i) at least twice daily; ii) once daily and iii) less than once daily, and for the RHINESSA study: i) never or rarely; ii) once per week; iii) once daily; iv) twice daily or v) more than two times per day. There was only one report (one participant) for each of the categories i) and ii) for the RHINESSA cohort. In the main analyses the categories were collapsed into “*<2 times per day”* and “*≥2 times per day”*.

**Analyses of mean CPI**

We assessed the association between FEV_1_/FVC ratio and CPI using the mean CPI-values per participant rather than the maximum value in order to investigate if the use of the maximum value could cause any loss of information and misclassification (S1 Table and S1 Figure).
