# Supplementary material for: Periodontal health status and lung function in two Norwegian cohorts
Source: PLoS One. 2018 Jan 19;13(1):e0191410. doi: 10.1371/journal.pone.0191410 (PMC5774767; doi:10.1371/journal.pone.0191410)
Supplement: S1 Table — (DOCX) [file pone.0191410.s004.docx]

S1 Table. Association between FV1/FVC-ratio and mean CPI, linear regression

|  | Model specification | n | Mean CPI, mean (min max) | B(95% CI) | p-trend |
| --- | --- | --- | --- | --- | --- |
| Model 9 | Adjusted for age and sex | 656 | 0.18 (0-3) | -0.015 (-0.028—0.001) | 0.031 |
| Model 10 | Adjusted for age, sex and smoking | 651 | 0.18 (0-3) | -0.012 (-0.025, 0.001) | 0.07 |
| Model 11 | Adjusted for age, sex, smoking, BMI, exercise, education | 645 | 0.18 (0-3) | -0.011 (-0.024, 0.003) | 0.11 |
| Model 12 | Adjusted for age, sex, smoking, BMI, exercise, education  Persons with asthma, asthma mediactio last 12 months or antibiotics last 12 months to help breathing excluded | 478 | 0.16 (0-2.3) | -0.013 (-0.025, -0.001) | 0.03 |
| Model 13 | As model 12 but with current smokers and former smokers excluded instead of adjustment for smoking | 308 | 0.11 (0-2.1) | -0.019 (-0.032, -0.006) | 0.004 |
